# A graphene-assisted all-pass filter for a tunable terahertz transmissive modulator with near-perfect absorption

**DOI:** 10.1038/s41598-019-49066-4

**Published:** 2019-08-29

**Authors:** Thang Q. Tran, Sangjun Lee, Sangin Kim

**Affiliations:** Department of Electrical and Computer Engineering, Ajou University, Suwon, 16499 South Korea

**Keywords:** Optoelectronic devices and components, Graphene

## Abstract

We proposed an all-pass filter based perfect absorber scheme which also can function as a highly efficient transmissive modulator. We theoretically analyzed the proposed scheme using the temporal coupled mode theory and showed that near-perfect absorption could be achieved with practically modest deviation from the critical coupling condition. We also demonstrated the feasibility of the proposed scheme in a grating-based all-pass filter device with a variable loss implemented by two separate graphene layers, achieving an absorption of ~99.8% and a transmission modulation depth of ~70 dB in a terahertz frequency range. We also numerically investigated the tunability of the designed device.

## Introduction

Recently, terahertz (THz) technology has been developed rapidly relying on the advances in source and detector development^1–4^ and there have been huge progress in its applications such as bio-medical imaging, security, time-domain spectroscopy, and communications^5^. In order to further develop the applications of the THz technology, the means to control and modulate the THz wave propagation should be developed, which, however, is still challenging since it is difficult to find materials responding to THz wave. To solve this problem, metamaterials, which are artificial structures enabling a variety of exotic electomagnetic properties, and their active control have been studied widely^6–12^. Recently, graphene has attracted a lot of interest as the means to enable the active control of the metamaterials due to its easily controllable permittivity via carrier density variation^13–20^. Moreover, an ultra-wide absorption bandwidth of graphene makes it considered as a promising candidate for a THz absorber. A myriad of studies to enhance the low absorption efficiency of atomically thin monolayer graphene have been conducted and many kinds of graphene-based THz perfect absorbers have been suggested^21–32^. In most of the previously suggested THz perfect absorbers, absorption enhancement is achieved by adopting a reflector as well as a resonant structure such as the metamaterial or a grating. Two representative types of the reflectors used in the previously THz perfect absorbers are a metallic mirror^21–29^ and a distributed Bragg reflector (DBR)^30–33^. Due to the aforementioned controllable permittivity of graphene, those perfect absorbers based on graphene can also work as modulators by introducing a proper capacitive structure to apply gate voltage to graphene, in which, however, only the reflected wave can be modulated because of the reflectors. So, if a perfect absorber can be realized without a reflector, a transmissive modulator of a high modulation depth is accompanied. In this work, we propose the reflectorless near-perfect graphene absorber scheme based on an all-pass filter composed of two identical gratings and numerically demonstrate ~99.8% absorption and its transmissive modulator operation with a ~70 dB modulation depth in a THz frequency range. We have theoretically investigated the operation principle of the proposed scheme using temporal coupled mode theory (TCMT)^34^. The numerical simulation was conducted using the rigorous coupled wave analysis (RCWA) method^35^ -based commercially available tool (DiffractMOD), and the particle swarm optimization (PSO) method^36^ was used for the device design.

## Results

### Theory

**Figure 1:**
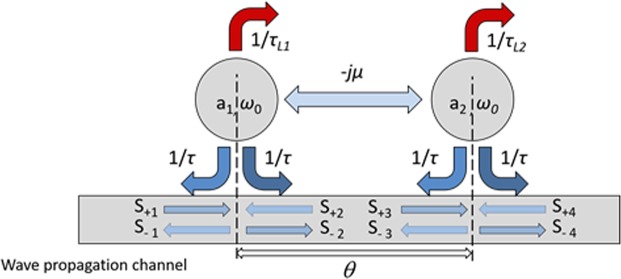
The schematic diagram of the coupled resonators. The two resonators are identical except having different loss rate 1/*τ*_*L*1_ and 1/*τ*_*L*2_. The resonators are assumed to be in all-pass filter mode when 1/*τ*_*L*1_ = 1/*τ*_*L*2_ = 0.

We performed the TCMT analysis for two lossy resonators with different loss rates of 1/*τ*_*L*1_ and 1/*τ*_*L*2_, but otherwise identical, having the same decay rates to the wave propagation channel 1/*τ*. The schematic diagram of the coupled resonators is presented in Fig. 1. The quality factor *Q* of the two resonators is related to the resonance frequency (*ω*_o_) and the decay rate 1/*τ* of the resonators through the relationship $Q={\omega }_{0}\tau /4$. The two resonators are coupled both indirectly through the propagation channel with a phase retardation of *θ* and directly through evanescent coupling with a coupling coefficient of *µ*. The temporal change of the normalized mode amplitudes of the two resonators (*a*_1_ and *a*_2_) can be described as follow:

1a $$\frac{d{a}_{1}}{dt}=(j{\omega }_{0}-\frac{1}{{\tau }_{L1}}-\frac{2}{\tau }){a}_{1}-j\mu {a}_{2}+\kappa {s}_{+1}+\kappa {s}_{+2},$$

1b $$\frac{d{a}_{2}}{dt}=(j{\omega }_{0}-\frac{1}{{\tau }_{L2}}-\frac{2}{\tau }){a}_{2}-j\mu {a}_{1}+\kappa {s}_{+3}+\kappa {s}_{+4},$$

where ${s}_{+i}$ and ${s}_{-i}$ are the complex amplitudes of the incoming and outgoing waves, respectively, and $\kappa$ is the coupling coefficient from the wave propagation channel to the resonators. Because of energy conservation and time reversal symmetry constraints, *μ* is real and $\kappa$ is given by $\kappa ={e}^{i\theta }\sqrt{2/\tau }$ ^34^.

In this work, we assumed that the all-pass filter conditions for the lossless case was satisfied, which are $\mu =2/\tau$ and $\theta =\pi /2$^37^. Assuming ${s}_{+4}=0$, and using the steady state condition with a time harmonic incident wave ${s}_{+1}=\overline{{s}_{+1}}{e}^{j\omega {\rm{t}}}$, we obtained the complex transmission and reflection coefficients of the system as follow:

2a $$t=\frac{\overline{{s}_{-4}}}{\overline{{s}_{+1}}}=j\frac{[4-\frac{{\tau }^{2}}{{\tau }_{L1}{\tau }_{L2}}+j(\omega -{\omega }_{0})\tau (\frac{\tau }{{\tau }_{L1}}+\frac{\tau }{{\tau }_{L2}})+{(\omega -{\omega }_{0})}^{2}{\tau }^{2}]}{[2+\frac{\tau }{{\tau }_{L1}}+j(\omega -{\omega }_{0})\tau ]\,[2+\frac{\tau }{{\tau }_{L2}}+j(\omega -{\omega }_{0})\tau ]},$$

2b $$r=\frac{\overline{{s}_{-1}}}{\overline{{s}_{+1}}}=j\frac{2\tau (\frac{1}{{\tau }_{L2}}-\frac{1}{{\tau }_{L1}})}{[2+\frac{\tau }{{\tau }_{L1}}+j(\omega -{\omega }_{0})\tau ]\,[2+\frac{\tau }{{\tau }_{L2}}+j(\omega -{\omega }_{0})\tau ]}.$$

From (2), it is found that perfect absorption (*r* = *t* = 0) is obtained only when ${\tau }_{L1}={\tau }_{L2}=\tau /2$, which implies the balance between the loss rate and the total leakage (coupling to the propagation channel) rate in each resonator and is the same as the critical coupling (perfect absorption) condition in the lossy one-port resonator with a reflector^38^. So, the perfect absorber based on the lossy all-pass filter can be understood as the variation of the lossy one-port resonator-based perfect absorber: the reflector is eliminated by placing another identical resonator with a mirror inversion symmetry. In another words, two resonators excited with the phase retardation of *θ* = π/2 is equivalent to placing a 100% mirror at the distance of λ/4 from one resonator (halfway between two resonators).

However, designing a system of two resonators with identical loss rate is quite difficult to achieve in practice. Therefore, we would like to analyze how a small deviation from the perfect absorption condition affect the absorption performance. Assuming the loss rate of the two resonators are given by

3 $$\{\begin{array}{c}{\tau }_{L1}=\frac{\tau }{2}+\delta /2\\ {\tau }_{L2}=\frac{\tau }{2}-\delta /2\end{array}$$

where $\delta \ll \tau$. At resonance ($\omega ={\omega }_{0}$), from (2) and (3), we obtain the approximated complex transmission and reflection coefficients:

4a $$t=j\frac{4-\frac{{\tau }^{2}}{(\frac{\tau }{2}+\frac{\delta }{2})(\frac{\tau }{2}-\frac{\delta }{2})}}{(2+\frac{\tau }{\frac{\tau }{2}+\frac{\delta }{2}})(2+\frac{\tau }{\frac{\tau }{2}-\frac{\delta }{2}})}\approx j\frac{4-\frac{{\tau }^{2}}{(\frac{\tau }{2}+\frac{\delta }{2})(\frac{\tau }{2}-\frac{\delta }{2})}}{16}\approx -\,j\frac{{\delta }^{2}}{4{\tau }^{2}},$$

4b $$r=\frac{2\tau (\frac{{\tau }_{L1}-{\tau }_{L2}}{{\tau }_{L1}{\tau }_{L2}})}{(2+\frac{\tau }{\frac{\tau }{2}+\frac{\delta }{2}})(2+\frac{\tau }{\frac{\tau }{2}-\frac{\delta }{2}})}\approx \frac{2\tau \frac{\delta }{{\tau }^{2}/4}}{16}=\frac{\delta }{2\tau }.$$

Therefore, when $\delta \ll \tau$, the transmission and the reflection can be approximated as $T={|t|}^{2}\simeq \frac{{\delta }^{4}}{16{\tau }^{4}}$ and $R={|r|}^{2}\simeq \frac{{\delta }^{2}}{4{\tau }^{2}}$, respectively. Since the transmission shows the fourth power dependence on the relative loss rate difference (*δ/τ*) near the perfect absorption condition, a very low transmission value can be obtained even for modest deviation from the perfect absorption condition, enabling a high transmission modulation depth with variation of the loss rate or the resonance frequency. The resulting absorption ($A=1-T-R\simeq 1-\,\frac{{\delta }^{2}}{4{\tau }^{2}}$) shows quadratic dependence on the relative loss rate difference ($\delta /\tau$) near the perfect absorption condition.

**Figure 2:**
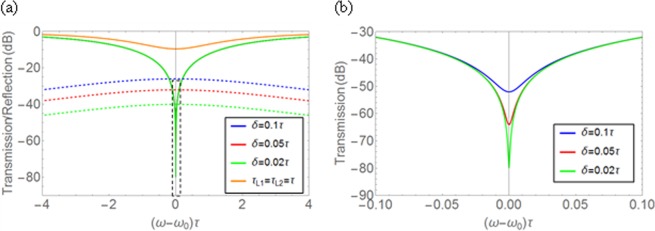
(**a**) Transmission (solid lines) and reflection (dashed lines) on a logarithmic scale as a function of frequency detuning with different deviation δ. (**b**) Enlarged version corresponding to the region represented by the dashed box in (**a**). Even a modest *δ* = 0.1*τ* (or about 20% of the loss of individual resonators) can result in a −50 dB minimum transmission. For non*-*zero *δ*, the all-pass filter condition is broken due to the difference of the resonators, resulting in non-zero reflection. On the contrary, when the loss coefficients are significantly far from the perfect absorption conditions, such as when *τ*_*L*1_ = *τ*_*L*2_ = *τ* (orange line), the maximum absorption is much less profound. It is noteworthy that in such case, since *τ*_*L*1_ = *τ*_*L*2_, the reflection coefficient is zero, and not shown in the figure.

Figure 2 shows the transmission and the reflection spectra calculated by (2) with (3) for several values of $\delta /\tau$ around the critical coupling condition (${\tau }_{L1}={\tau }_{L2}=\tau /2$). The case far away from the critical coupling condition (${\tau }_{L1}={\tau }_{L2}=\tau$), corresponding to the case that the loss is the half of the critical coupling condition and *δ* = 0, is also plotted for reference, which is represented by the orange curve. Note that when the loss difference between two resonators is introduced under the non-critical coupling condition such as ${\tau }_{L1}={\tau }_{L2}=\tau$, the minimum transmission value rather increases slightly because the absorption decreases with the critical coupling condition further ruined by the loss imbalance. So, we conservatively adopted the *δ* = 0 case for reference. For non-zero *δ* around the critical coupling condition, the all-pass filter condition is slightly broken due to the difference between two resonators, resulting in non-zero reflection. However, the reflection is acceptably small, which is ~−30 dB at the resonance even for *δ* = 0.1*τ*. Fig. 2(b) is the enlarged graph corresponding to the region represented by the dashed box in Fig. 2(a). As expected, a reasonable 20% loss difference (*δ* = 0.1*τ*) around the critical coupling condition still resulted in quite low transmission of ~−50 dB at the resonance frequency. So, if we can somehow change the losses of resonators from ${\tau }_{L1,2}=\tau \pm \delta /2$ to ${\tau }_{L1,2}=\tau /2\pm \delta /2$, a transmission modulation depth of ~40 dB can be achieved. For 4% loss difference (*δ* = 0.02*τ*), the minimum transmission of ~−80 dB and a modulation depth of ~70 dB can be achieved. If the loss variation is increased by choosing the smaller loss close to zero (${\tau }_{L1,2}\, \sim \,\infty$) for the high transmission state (reference), the larger modulation depth can be obtained in each case. The estimated absorptions at the resonance frequency are ~99.9% and ~99.99% for the moderate loss difference values of 20% and 4%, respectively.

The loss in the TCMT modeling is the summation of all kinds of losses such as material absorptions and scattering/radiation losses of the resonators:

5 $$\frac{1}{{\tau }_{L}}=\frac{1}{{\tau }_{absorption}}+\frac{1}{{\tau }_{scattering}}+\frac{1}{{\tau }_{radiation}}=\frac{1}{{\tau }_{variable}}+\frac{1}{{\tau }_{background}}$$

where the background loss represents all the fixed losses that we cannot control. In the ideal case of no background loss, the variable loss can be changed from zero to 2/τ to achieve a maximum transmission modulation depth. If there is an unwanted background loss, the minimum loss cannot be zero, so that the minimum transmission value of the high state will somewhat decrease, but the low transmission state can be the same as the ideal case by choosing the variable loss to make the total loss 2/τ. The high transmission state (reference) considered in Fig. 2 corresponds to the case of 1/τ_*backgroud*_ = 1/τ, in which still a considerably high modulation depth can be achieved because the proposed transmissive modulator scheme is based on the low state of the near perfect absorption resulted from the critical coupling condition. Note that the performance (or the minimum transmission value) of the low state relies on the balance between the total loss and the leakage, not the absolute value of 2/τ, which is associated to the quality factor of the resonator (Q = ω_ο_τ/4). The only constraint for the high modulation depth with near perfect absorption is that the background loss should be smaller than 2/τ. Our TCMT modeling reveals that a background loss rate of 1/τ can achieve a considerably high modulation depth of ~70 dB for a 4% loss difference between two resonators in the proposed scheme.

### Near-perfect absorption and transmission modulation in grating based structure

**Figure 3:**
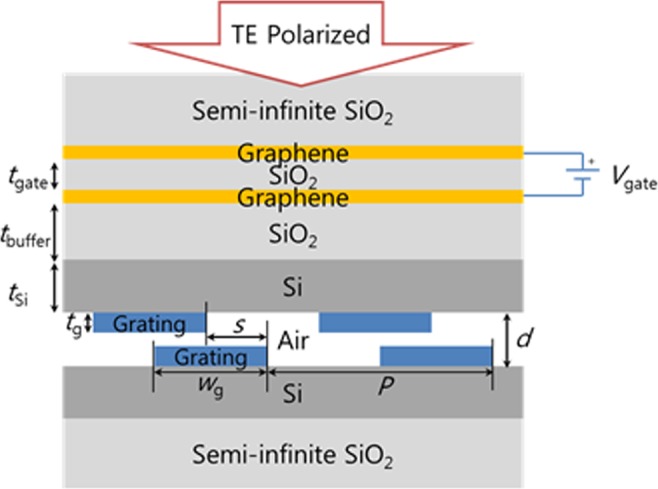
Schematic of the grating-based graphene-assisted all-pass filter THz modulator. Functionally, the structure can be divided into two parts: the grating-based all-pass filter, and the graphene absorbing layers. The gratings made of Si or PDMS were considered. In our designs, the fill factor *FF* is fixed at 0.5 and *t*_gate_ = 7 nm. To obtain an operating wavelength around 50 µm, the optimal design parameters for the Si grating-based device, unless otherwise indicated, were selected as follows: *t*_g_ = 0.593 µm, *P* = 18.775 µm, *t*_Si_ = 2.67 µm, *d* = 1.486 µm, *s* = 7.703 µm and *t*_buffer_ = 8 µm.

Based on the theory described in the previous section, we designed a terahertz near-perfect absorber based on a graphene-assisted all-pass filter. The schematic diagram of the device is presented in Fig. 3, where two identical gratings form the all-pass filter, and the graphene layers are added to introduce loss. The-all pass filter consists of two gratings with thickness *t*_g_, a period *P*, and a fill factor *FF* (=*w*_g_/*P*), formed on a Si layer of thickness *t*_Si_ and separated by an air gap with a distance *d*. The gratings made of Si or PDMS were considered in this work. One device with Si gratings was designed, having a quality factor of ~10^3^ and the other with PDMS gratings was designed to have a much higher quality factor of ~10^5^. The direct coupling coefficient and the phase retardation in this device are controlled by *d* and a lateral shift distance *s*^39^. Two graphene layers separated by a thin SiO_2_ layer with a thickness *t*_gate_ are embedded in SiO_2_. The graphene absorbing layers and the all-pass filter are separated by a distance *t*_buffer_. To tune the loss via variation of the complex permittivity of graphene, a gate voltage *V*_gate_ is applied between the two graphene layers. The complex permittivity of graphene was calculated using the Kubo formula^40^ with following assumptions: the thickness of graphene was 0.34 nm, the Fermi velocity *V*_F_ was 10^6^ m·s^−1^, and the mobility *μ* was 1000 cm^2^·V^−1^·s^−1^. The permittivity of Si and SiO_2_ were assumed to be 3.4167^2^ and 2.1269^2^, respectively.

In our design, the structural parameters denoted in Fig. 3 were optimized first to realize the all-pass filter without the graphene layers at an operating wavelength around 50 µm, and then, re-optimized to minimize the transmission at the resonance with the graphene layers, obtaining the near-perfect absorber design. Note that the introduction of the graphene layers not only adds loss but also changes the all-pass filter condition slightly. The numerical simulation was conducted using the RCWA method^35^ -based commercially available tool (DiffractMOD), and the PSO method^36^ was used for the device design. In our design process, the grating fill factor and the gap between the graphene layers were fixed at *FF* = 0.5 and *t*_gate_ = 7 nm. When the chemical potential (Fermi level) of graphene is 0.1521 eV, the optimal design parameters for the device with Si grating were found as follows: *t*_g_ = 0.593 µm, *P* = 18.775 µm, *t*_Si_ = 2.67 µm, *d* = 1.486 µm, *s* = 7.703 µm, and *t*_buffer_ = 8 µm. In the rest of this paper, these parameters are used unless otherwise indicated. Because *t*_buffer_ is much larger than *d*, the loss rates of the two resonators due to the presence of the graphene layers would be very close, causing the structure to operate in the near-perfect absorber mode. One can easily decrease both *τ*_*L*1_ and *τ*_*L*2_ simultaneously simply by increasing *t*_buffer_, and there should be a proper *t*_buffer_ where the near-perfect absorption condition is satisfied. Therefore, simply by changing *t*_buffer_, one should be able to find a point where nearly perfect absorption occurs without modifying parameters of the all-pass filter structure. Likewise, one could also change both *τ*_*L*1_ and *τ*_*L*2_ simultaneously by changing the gate voltage applied between the graphene layers, which causes the change of the chemical potential of graphene.

**Figure 4:**
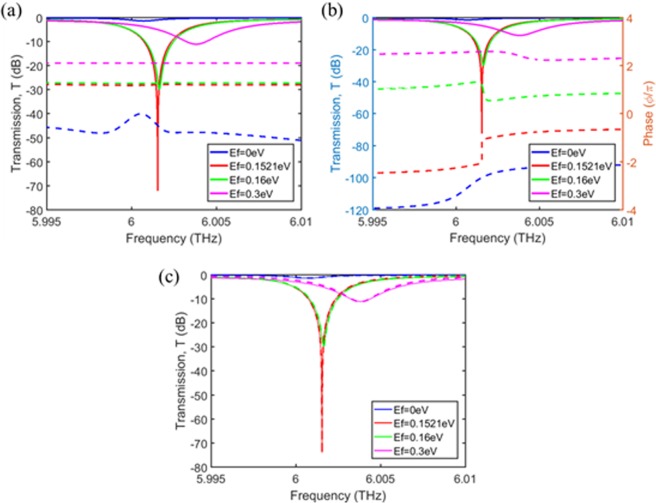
Spectral response of the device for various chemical potentials of graphene. The nearly perfect absorption point is *E*_f_ ≈ 0.1521 eV. (**a**) The transmission (solid line) and reflection (dashed line) of the device. (**b**) The transmission (solid line) and phase shift (dashed line) of the device. (**c**) Comparison between the numerical calculation (solid line) and the TCMT modeling (dashed line).

Figure 4(a) shows the calculated transmission (solid lines) and reflection (dashed lines) spectra of the designed device for various chemical potentials of graphene. When *E*_f_ = 0.1521 eV, the near-perfect absorption is achieved: the transmission dip is ~−70 dB is at the resonance and the reflection is ~−30 dB, resulting in an absorption of ~99.84%. When *E*_f_ = 0 eV, which corresponds to the case of almost the same and relatively low losses for two resonators, the reflection is as low as ~−50 dB, but the transmission is quite high, so that we obtain low absorption. This implies that we can achieve a transmission modulation depth of ~70 dB by changing the chemical potential from *E*_f_ = 0 eV (the high transmission state) to *E*_f_ = 0.1521 eV (the low transmission state). When the chemical potential is varied slightly around the optimal value, the resonance frequency variation is negligible, but the minimum transmission increases considerably due to the broken critical coupling condition resulting from the loss rate change. One can see that the minimum transmission increases up to ~30 dB for *E*_f_ = 0.16 eV. When the chemical potential is increased to 0.3 eV, the loss of the graphene layers decreases way below the *perfect* absorption condition and the transmission becomes rather high with low absorption. At the same time, the reflection also increases due to the enhanced conducting property of highly doped graphene layers while the relative difference in the loss rates of two resonators is kept about the same. Since the variation of the chemical potential changes both the real and the imaginary parts of the graphene permittivity, the shift of the resonance (minimum transmission) frequency is observed when *E*_f_ varies. Figure 4(b) shows the phases of the transmitted waves for various chemical potentials. The abrupt phase change at resonance is observed when the near-perfect absorption condition is achieved (*E*_f_ = 0.1521 eV), indicating very strong resonance of the all-pass filter despite the presence of resonator losses. Figure 4(c) shows the comparison between the numerical (RCWA) calculation and the TCMT modeling given in (2a), where one can see excellent agreement. The parameters used in the TCMT modeling are listed in Table 1. As the graphene chemical potential varies, the resonance frequency (ω_o_) and the loss rate (1/τ_L_) change as expected. Whereas, it appears that the leakage rate of the resonator (1/τ) is mainly determined by the grating structure, not by the graphene chemical potential for *t*_buffer_ = 8 µm. The relative loss rate difference (δ/τ) is not affected by the chemical potential change either, which seems to be closed related to the location of the graphene layers (*t*_buffer_).

**Table 1:** Parameters used in the TCMT modeling shown in Fig. 4(c).

Parameters	ω_0_ (THz)	τ (ps)	τ_loss_ (ps)	δ
**E**_**f**_				
0 eV	6.000717	1666	22222	0.0288
0.1521 eV	6.001547	1666	1666	0.0288
0.16 eV	6.001643	1666	1563	0.0288
0.3 eV	6.003821	1666	939	0.0288

**Figure 5:**
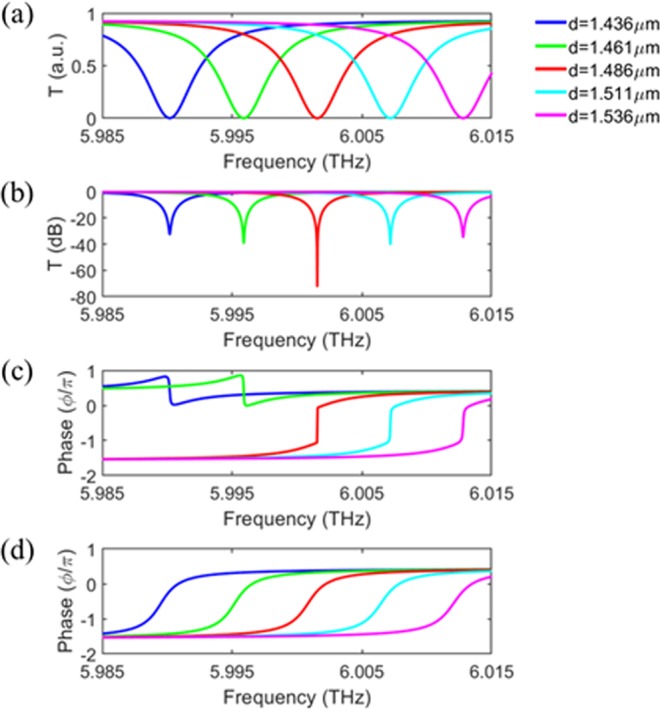
Transmission and phase shift of the device when the air gap distance (*d*) changes, while all the other parameters remain constant. The transmission vs wavelength was plotted on a linear scale (**a**), and a logarithmic scale (**b**), and the phase shift was plotted in (**c**). The phase shift of the all pass filter structure without the graphene layers was plotted in (**d**) for reference. This data demonstrated the ability of the device to tune the resonance wavelength by simply changing the air gap distance.

We also investigated the resonance frequency tunability of our device. Figure 5(a,b) show the calculated transmission spectra on linear and logarithmic scales, respectively, for various air gap distances (*d*) with all the other parameters kept the same. Quite reasonable performance of ~−30 dB minimum transmission can be achieved while the operating frequency is fine tuned. Figure 5(c,d) show the phase of the transmission with and without the graphene layers. One can see that this resonance frequency tunability is inherent to the grating based all-pass filter structure, and not a consequence of the near-perfect absorber design presented in this work.

**Figure 6:**
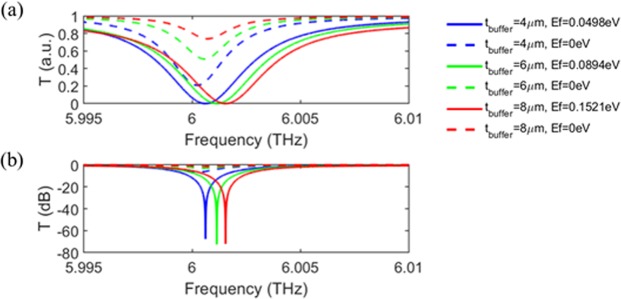
Transmission of the device when *t*_buffer_ changes on a linear scale (**a**) and a logarithmic scale (**b**), at the corresponding critical coupling chemical potential (solid line) and zero chemical potential (dashed line) of graphene. There is a trade-off between the insertion loss and the chemical potential change required for the full transmission modulation. There is some frequency tuning effect but not quite significant compared to simple tuning of the air gap distance discussed above.

Figure 6 shows the transmission modulation performance for various t_buffer_, where the trade-off between the insertion loss and the chemical potential change required for the full transmission modulation. When the graphene layers are closer to the all-pass filter, the loss is higher and thus, the insertion loss for the high transmission state is higher (*E*_f_ = 0). At the same time, the optimal graphene loss coefficient to satisfy the critical coupling condition decreases because the field confinement in the graphene layers increases while the quality factor of the all-pass filer remains the same. Note that the location of the graphene layers does not affect the quality factor of the all-pass as mentioned earlier. Then, the chemical potential change for the full transmission modulation becomes smaller. Note that the loss of graphene increases as the chemical potential increases for a wavelength longer than ~10 μm^40^. So, in terms of the insertion loss, the larger t_buffer_ is desirable, which, in return, increase the chemical potential variation required for the full transmission modulation. If the insertion loss below 1.0 dB (that is, *T* = ~80% at *E*_f_ = 0 eV) is required, according to our investigation, the optimal condition appears to be *t*_buffer_ = ~8 μm and the low transmission state of *E*_f_ = ~0.1521 eV.

## Discussion

In this work, we have provided the theoretical framework of a lossy all-pass filter based near-perfect absorber. Based on this, we have designed a graphene-assisted all-pass filter, which is composed of two identical gratings, for a tunable THz transmissive modulator with near-perfect absorption, and numerically demonstrated an absorption of ~99.8% and a transmissive modulation depth of ~70 dB via graphene chemical potential variation of ~0.15 eV.

**Figure 7:**
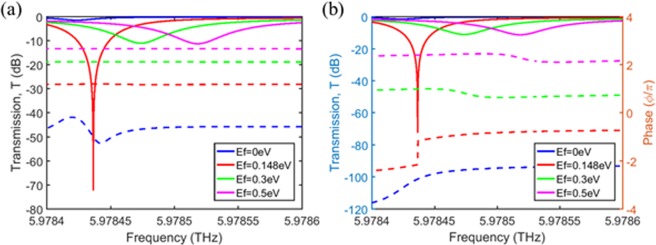
Spectral response of the device for various chemical potentials of graphene when the PDMS gratings are used. The nearly perfect absorption point is *E*_f_ ≈ 0.148 eV. (**a**) The transmission (solid line) and reflection (dashed line) of the device. (**b**) The transmission (solid line) and phase shift (dashed line) of the device.

In our design of the transmissive modulators in the previous section, the gratings were assumed to be made of Si for the convenience of simple design, which has a modest quality factor of ~10^3^. A grating structure with a quality factor of ~10^3^ was fabricated and a ~80% guided-mode resonance (GMR) reflection peak at λ = 742.5 nm was experimentally demonstrated^41^, corresponding to a background loss of ~0.25/τ. This implies that the constraint for the high modulation depth with near perfect absorption (a background loss smaller than 2/τ) can be met in a practical grating with a quality factor of ~10^3^. Moreover, ~95% GMR reflection peak of ~700 quality factor at λ = ~1550 nm (corresponding to a background loss of ~0.06/τ) was fabricated^42^, where authors claimed that the high reflection peak was achieved via fabrication process improvement. This implies that the dominant source of the background loss in gratings is not the material loss, but the scattering loss due to the imperfect fabrication. Assuming an extremely advanced fabrication technology development, a device with a much higher quality factor can be designed with a modified grating structure, as well. As an example, we designed the device with the gratings made of PDMS (*n* = 1.5290). When the chemical potential of graphene is 0.148 eV, the optimal design parameters were found as follows: *t*_g_ = 0.63001 µm, *P* = 19.92114 µm, *t*_Si_ = 2.49017 µm, *d* = 1.50903 µm, *s* = 8.327 µm, and *t*_buffer_ = 22.2 µm. Due to the decreased leakage rate, the graphene layers are located farther away from the all-pass filter to satisfy the critical coupling condition. As seen in Fig. 7, the designed transmissive modulator shows a much higher quality factor close to 10^5^.

In the fabrication of the proposed device, the realization of two identical gratings is quite important. One possible way to realize two identical gratings is to halve a single large grating and put them together facing each other. The Si grating structure can be fabricated on a Si substrate and put on the SiO_2_ (glass) substrate after the Si substrate is thinned down to the designed thickness. One of the SiO_2_ (glass) substrate should be prepared to have two graphene layers, which can be conducted with a standard graphene transfer process and SiO_2_ depositions using an atomic layer deposition (ALD) and a low-pressure chemical vapor deposition (LPCVD) processes.

The proposed transmissive modulator with a high modulation depth can be used in terahertz communications. Since a signal-to-noise ratio after passing through a modulator is directly related to the modulation depth of the modulator, our high modulation depth device will be quite useful in terahertz communication applications. Besides, the resonance frequency of our device can be sensitively tuned by the air gap distance change, which implies the refractive index change in the gap will also change the resonance frequency. This feature can be exploited for bio-chemical sensor applications. As shown in Fig. 7, the quality factor of our device can be achieved, which will result in a highly sensitive sensor.

Our proposed scheme can be applied to other types of all-pass filter realized by photonic crystal^43^, ring resonator^44^, or planar types^45^, potentially opening a new class of devices capable of operating in a wide variety of environments. Since the critical coupling condition is relaxed, as long as the all-pass filter condition can be achieved, by tuning the loss rate, we can potentially achieve near-perfect absorption at any wavelength desired.

## Methods

To theoretically investigate the operation principle of the proposed scheme, we used the temporal coupled mode theory (TCMT)^34^. The numerical simulation was conducted using two-dimensional RCWA (a commercial software, DiffractMOD)^35^, where more than 300 harmonics were applied to guarantee accuracy near the resonant frequency. For the optimal device design, the particle swarm optimization (PSO)^36^ method was applied. In all RCWA calculations, the complex permittivity of graphene (*ε*_*g*_) was calculated using Kubo formulation based on the local random phase approximation for various *E*_*f*_^40^, assuming graphene thickness of 0.34 nm, Fermi velocity of 10^6^ m/s, and mobility of 0.1 m^2^/Vs.
